# Effect of Receptor Dimerization on Membrane Lipid Raft Structure Continuously Quantified on Single Cells by Camera Based Fluorescence Correlation Spectroscopy

**DOI:** 10.1371/journal.pone.0121777

**Published:** 2015-03-26

**Authors:** Heng Huang, M. Fethullah Simsek, Weixiang Jin, Arnd Pralle

**Affiliations:** 1 Department of Physics, University at Buffalo the State University of New York, Buffalo, New York, United States of America; 2 Department of Biophysics and Physiology, University at Buffalo the State University of New York, Buffalo, New York, United States of America; German Cancer Research Center, GERMANY

## Abstract

Membrane bound cell signaling is modulated by the membrane ultra-structure, which itself may be affected by signaling. However, measuring the interaction of membrane proteins with membrane structures in intact cells in real-time poses considerable challenges. In this paper we present a non-destructive fluorescence method that quantifies these interactions in single cells, and is able to monitor the same cell continuously to observe small changes. This approach combines total internal fluorescence microscopy with fluorescence correlation spectroscopy to measure the protein’s diffusion and molecular concentration in different sized areas simultaneously. It correctly differentiates proteins interacting with membrane fences from proteins interacting with cholesterol-stabilized domains, or lipid rafts. This method detects small perturbations of the membrane ultra-structure or of a protein’s tendency to dimerize. Through continuous monitoring of single cells, we demonstrate how dimerization of GPI-anchored proteins increases their association with the structural domains. Using a dual-color approach we study the effect of dimerization of one GPI-anchored protein on another type of GPI-anchored protein expressed in the same cell. Scans over the cell surface reveal a correlation between cholesterol stabilized domains and membrane cytoskeleton.

## Introduction

Many forms of cell membrane bound signaling require the interaction of diffusing membrane proteins, such as dimerization of or kinase activity on a receptor. These interactions are likely modulated by the two main membrane ultra-structure elements[1–7]. Some diffusing proteins are corralled between “fences” created by cytoskeleton-anchored membrane-associated proteins[8]; other diffusing proteins are transiently captured or trapped in either protein nanoclusters or cholesterol-dependent lipid nanodomains, so-called lipid rafts[2,3,9]. Both structures are too small and too dynamic to be directly imaged by optical microscopy. Thus far, the methods used to characterize lipid domains in live cells come with limitations: fluorescent labeling of lipids (e.g. with Cholera toxin B or antibody) [10] may perturb the domains; single particle tracking, thermal noise imaging, and homo-FRET measurements [11–13] are technically extremely challenging; Super-resolution imaging (PALM, STORM) and image correlation microscopy [14] are currently limited to more static structures due to their temporal resolution. Additionally, most of these require averaging over multiple cells or areas of cells, which may vary widely due to cell cycle, substrate adhesion, or other still unknown factors. Most importantly, none of these methods is able to continuously measure the protein-membrane interactions in single cells with sufficient resolution and provide enough statistics to observe the dynamic changes caused by external parameters, stimuli, or cell signaling. Such continuous spatially resolved observation on single cells is absolutely critical for the study of dynamic signaling or drug-induced perturbations.

We present a simple, nondestructive method capable of continuously monitoring the interaction of fluorescently tagged membrane proteins or lipids with the membrane ultra-structure. This capability permits us to study the time-course changes of protein-domain association in response to ligand induced dimerization, temperature, or perturbations caused by drug induced changes to the cytoskeleton. This method is sensitive to small differences in the ectodomain which may affect protein dimerization, as between enhanced-GFP and monomeric-GFP. Our method utilizes spatially resolved camera based fluorescence correlation spectroscopy (FCS) [15] to record membrane protein diffusion on multiple length scales simultaneously.

Confocal FCS has been widely used to measure membrane protein diffusion, showing the diffusion to be anomalous [16] and deviating from free Brownian motion. In 2005, Wawrezinieck et al. [17] performed multiple FCS measurements with increasing beam waist *ω* and analyzing the relationship between the transit time through the beam *ω* ^2^. They proposed it would be possible to distinguish three different diffusion modes and their respective underlying membrane ultra-structures: (**i**) free Brownian diffusion in a homogenous environment; (**ii**) diffusion slowed by transient trapping in sub-microscopic protein or lipid domains; or (**iii**) diffusion hindered by interaction with a grid of diffusion barriers, i.e. ‘membrane fences’. Using confocal FCS to collect data for this analysis requires multiple, sequential measurements with a series of excitation volumes and calibration of each of these volumes, thereby prohibiting the investigation of the time-course of dynamic events.

By contrast, using a camera to measure FCS [15] enables us to bin the signal obtained in each camera pixel into binned-pixels covering a range of sizes. We refer to this method as binned-imaging FCS (bimFCS) [18]. Because bimFCS measures the transit times through differently sized areas simultaneously, it enables us to study dynamic signaling events in single cells. Unlike confocal FCS, bimFCS does not require experimental calibration because the observation area of each pixel is fixed (see SI Methods). After our initial proposed method [18], others have presented camera based correlation methods to study the interaction of lipids with cholesterol-stabilized domains [19]. For example, the Wohland group recently demonstrated that the method may be used to analyze the temperature dependence of lipid diffusion in bilayers and cells [20]. However, none of the other studies were able to follow the fate of a single cell over time while perturbing its receptors. Further, the TIRF FCS studies had insufficient temporal and spatial resolution to obtain nanodomain interaction times consistent with confocal and STED studies [20]. In this study, we present the first continuous observation of single cells resolving the effect of receptor dimerization on lipid rafts. Our results agree with the confocal and STED FCS data both qualitatively and quantitatively [20]. In addition, we are able to provide the first result on non-raft proteins interacting with the membrane cytoskeleton.

## Materials and Methods

Information on cells and experiment conditions, transfection DNA plasmids for fluorescently labeled proteins, details of chemical treatments, and specificity of monoclonal antibodies can be found in the Supporting Material.

### Instrumental Setup

Lines from an Argon-Krypton laser (Innova 70C, Coherent) are fiber-coupled into an inverted microscope (AxioObserver, Zeiss), focused on the back focal plane of the objective lens (Zeiss, 100x oil, NA = 1.45) and adjusted to achieve TIRF angle. The illuminated TIRF spot in the image plane is 515*mm* ^2^ (see SI for details). Waist of the point spread function (PSF) of the optical setup was measured to be σ = 108.8*nm* (525/39nm), σ = 130.5*nm* (593/40nm), and σ = 117.5*nm* (590/20nm) for different filter sets used. A laser power of 3*μW* at the objective lens (582.5 *W/m* ^2^ in the sample plane) was used for GFP-labeled proteins. Under this illumination, photo bleaching within the each pixel is negligible, yet the signal-to-noise is sufficient (see S3*c* Fig. for effect of excitation power on bimFCS results). Fluorescence signals from the bottom membrane of the cell (or lipid bilayer) are collected by the objective, filtered and acquired by an EMCCD (Andor iXon^+^ 897) that is controlled by the Andor Solis software. The area of the image plane covered by each camera pixel is adjusted by placing a lens of appropriate magnification in front of the camera and by on-camera pixel binning. The pixel sizes used here are *64 nm x 64 nm* and 160 *nm ×* 160 *nm* for intact cells and lipid bilayer respectively.

### Data analysis

**Fig 1:**
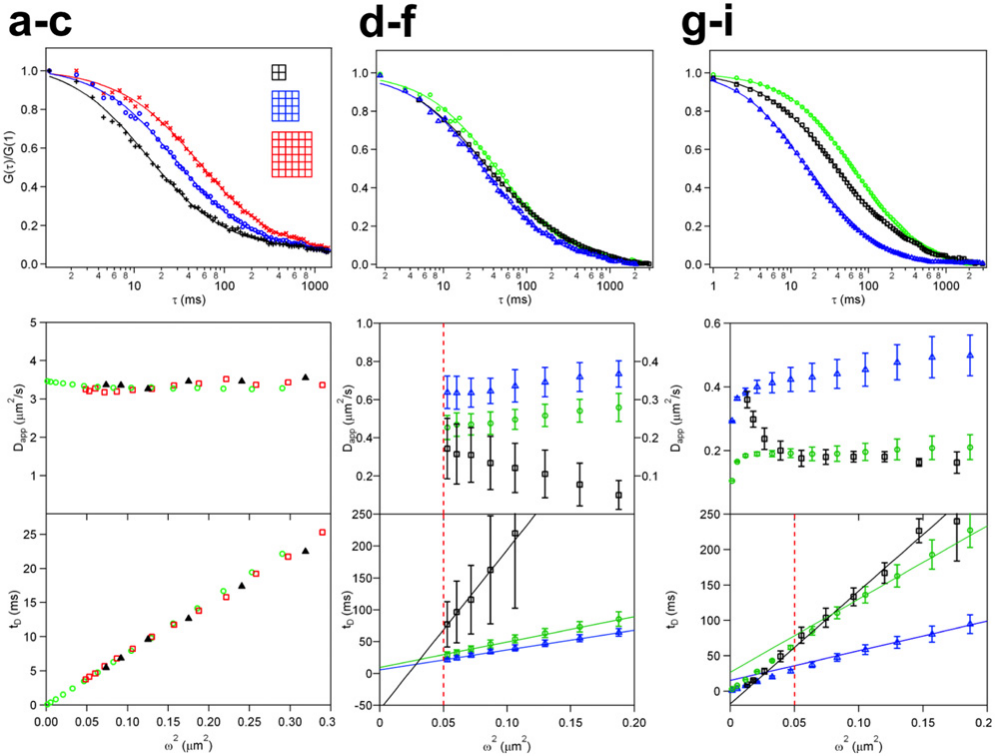
Principle of bimFCS analysis, FCS curves and bimFCS analysis of differently anchored membrane proteins. (A) Normalized FCS curves for several pixel-bin sizes (bin 2, 4 and 6) from LissRhod PE-lipids freely diffusing in a supported lipid bilayer (SLB) with LissRhod PE:DOPC ratio of 1:10000. (B) Apparent diffusion coefficients (*D*_*app*_, top) and the corresponding transit time *t*_*D*_ (bottom) are plotted versus observation area *ω* ^2^ for LissRhod PE in a SLB (black, filled triangles, *N* = 9) and overlaid with results from two Monte-Carlo Simulations. One simulation considers the microscope optics by convoluting the pixels with the PSF (red, empty squares), while the other one is analyzed as super-resolution (SR) without convoluting with the PSF (green, empty circles). (C) Comparison of the normalized FCS curves (*ω* ^2^ = 0.087 *μm* ^2^) obtained from mGFP-GPI (green, empty circles), mGFP-GPI with COase treatment (blue, empty triangles) and mGFP fused LDL-R transmembrane domain (mGFP-TM; black, empty squares) in PtK2 cells at 37°C. Data from GPI-anchored and LDL-R anchored mGFP proteins is fitted with one and two diffusion-component square-pinhole FCS functions respectively. (D) *D*_*app*_ (top) and the corresponding *t*_*D*_ (bottom) versus *ω* ^2^relationships of mGFP-GPI (green, empty circles), mGFP-GPI with COase treatment (blue, empty triangles) and mGFP-TM (black, empty squares) in PtK2 cells. A linear fit through the *t*_*D*_ versus *ω* ^2^relationship obtained from mGFP-GPI (*N* = 9) yields a time-axis intercept *t*_0_ of 12±4 *ms* whereas mGFP with transmembrane anchor (*N* = 3) gave a negative time-axis intercept, *t*_0_ = -60±10 *ms*. In cells whose cholesterol has been oxidized using COase (*N* = 11), *t*_0_ is decreased to be 7±2 *ms*. (E) Normalized FCS curves (*ω* ^2^ = 0.053 *μm* ^2^) of Kinetic Monte-Carlo simulations for diffusers transiently trapped in submicroscopic domains (green, empty circles, *d* = 16%,*l* = 30*nm*), weakly interacting with such domains (blue, empty triangles) or interacting with and hopping over fences (black, empty squares, *a* = 75*nm*). Data for fence simulation is fit globally using two-component square-pinhole FCS function with held free diffusion coefficient for the fast component of the curve. (F) *D*_*app*_ (top) and the corresponding *t*_*D*_ (bottom) versus *ω* ^2^ relationships obtained from SR analysis of simulations for diffusers transiently trapped in submicroscopic domains (green, empty circles), weakly interacting with such domains (blue, empty triangle) or interacting with fences (black, empty squares) (*N* = 3). Linear fit of the *t*_*D*_ versus *ω* ^2^ relationship beyond optically accessible size (red, dashed line) for transiently trapping simulations yields a time-axis intercept *t*_0_ of *27 ms* for strong and 15 *ms* for weak interaction simulations. Linear fit for the results of fence simulations showed a negative time-axis intercept t_0_ = -16 *ms*.

All data analysis was performed using custom written software routines in Igor Pro (available upon request; see S2 Fig. for a flowchart of the data analysis). Stacks of 16-bit fluorescence images are loaded into a 3-D intensity matrix. As the TIRF illumination area is significantly larger than the pixels used for FCS, photo bleaching causes a loss of fluorophores during continuous data acquisition, which is mathematically compensated (See SI Text and S3 Fig. for details). For each bin size n, the intensity values within each n × n pixel box are averaged. Further, bin n pixels are generated by sliding the box half the box length at a time until covering the ROI. The FCS curve of each binned unit was calculated and then averaged over the ROI. The averaged FCS curve for each bin size is fitted (Fig. 1b) with square-pinhole FCS function for lateral diffusion [21]:

$$\begin{array}{l} G\left(\tau \right)=G_{\infty }+\frac{1}{N}{\left(\frac{2}{a^{2}\sqrt{\pi }}\sqrt{\sigma^{2}+D_{app}\tau }\left(\exp\left(-\frac{a^{2}}{4\left(\sigma^{2}+D_{app}\tau \right)}\right)-1\right)+\frac{1}{a}\text{erf}\left(\frac{a}{2\sqrt{\sigma^{2}+D_{app}\tau }}\right)\right)}^{2}/\\ {\left(\frac{2\sigma }{a^{2}\sqrt{\pi }}\left(\exp\left(-\frac{a^{2}}{4\sigma^{2}}\right)-1\right)+\frac{1}{a}\text{erf}\left(\frac{a}{2\sigma }\right)\right)}^{2} \end{array}$$ (1)

to obtain *D*_*app*_ value and the average number of diffusing molecule *N* for the corresponding bin size. Here *a* is the side length of binned pixel and σ the width of PSF. The molecular density for each bin can be calculated as *C* = *N*/*πω* ^2^ and averaged over different bin sizes with *ω* ^2^ ranging between 0.07–0.25 *μm* ^2^. Reliability of molecular concentration values are tested using supported lipid bilayers with known concentrations and in simulations (S5 Fig.). The transit time *t*_*D*_ for each bin is calculated using Einstein’s diffusion law, *ω* ^2^ = 4*D*_app_ *t*_*D*_, and plotted against *ω* ^2^. The resulting *t*_*D*_ versus *ω* ^2^ curve is then fitted with a straight line (through data points with *ω* ^2^ values greater than 0.07 μm^2^ and *t*_*D*_ values smaller than 1/1000 of the total length of data analyzed) to obtain the effective diffusion coefficient *D*_*eff*_ and time-axis intercept *t*_0_. For observing dynamic changes in single cells, continuously acquired bimFCS data was analyzed in 60s segments with 30s intervals. The molecular concentration *C* effective diffusion coefficient *D*_*eff*_, and time-axis intercept *t*_0_ are calculated for each time point as described above. The bleach-corrected and normalized molecular concentration *C*_*norm*_ is calculated by fitting the time-varying *C* values before treatment (t<0) with an exponential decay function and dividing the original *C* values by the resulting fit function.

## Results

### Simultaneous multi-length scale diffusion measurements by bimFCS

To validate our camera FCS data acquisition and analysis, we measured the diffusion of the fluorescently labeled lipid Liss Rhod PE (lissamine rhodamine B sulfonyl labeled phosphatidyl-ethanolamine) diffusing in a supported DOPC lipid bilayer (on glass, see SI for details), and performed Monte-Carlo Simulations of two-dimensional free diffusion. For data taken from the same bilayer, normalized FCS curves of different binned-pixels show that the autocorrelation time increases with increasing bin size, as expected for a diffusion-dominated process (Fig. 1*a*). The apparent diffusion coefficient (*D*_*app*_) obtained from PE lipids in a lipid bilayer is independent of the observation area *ω* ^2^ (Fig. 1*b*, top), and the *t*_*D*_ versus *ω* ^2^ relationship falls on a straight line through the origin (Fig. 1*b*, bottom), as expected for free Brownian diffusion. Two simulations of two-dimensional free diffusion analyzed by bimFCS (see SI Simulation Details) were overlaid on the experimental data (Fig. 1*b*): a super-resolution simulation using a *δ*-function as point-spread function (PSF) and *ω* ^2^ set as equal to the pixel area divided by *π*, and a simulation using the PSF of the microscope and computed equivalent *ω* ‘s for the binned pixels. The perfect overlap of the super-resolution and PSF-convoluted simulations verifies our method of computing equivalent beam waists *ω* for each bin size (See SI for computation method). The good agreement between the simulations and experimental data confirms bimFCS’s capability to identify free diffusion in a homogeneous medium.

### Comparison of bimFCS results of differently anchored membrane proteins in intact cells

The plasma membrane is heterogeneous and the apparent diffusion coefficient of membrane proteins depends on the observation area [22]. Transient trapping in sub-resolution domains results in slower local diffusion [2,3], while hopping over membrane fences reduces long-range diffusion [5, 23]. To show how these effects on protein diffusion in the cell membrane would manifest themselves in our method, we observed the diffusion behavior of fluorescent proteins fused to raft markers (GPI anchor) or transmembrane (LDL-R) domains.

Fig. 1*c* compares the normalized FCS curves calculated for the same binned-pixel size (*ω* ^2^ = 0.087 *μm* ^2^) and averaged over the ROI, for diffusion of monomeric GFP (mGFP) molecules deviating differently from free diffusion behavior. The FCS curves of the GPI-anchored mGFP (mGFP-GPI) in cells treated with cholesterol oxidase to dissolve cholesterol-stabilized domains (blue), and in untreated cells (green) are well described by free diffusion, the only difference being that the data after oxidization of membrane cholesterol (blue) shows a shortened diffusion transit time. The oxidization of the cholesterol is expected to destabilize the domains [24, 25]. However, the correct identification of protein interaction with submicroscopic domains is not possible with single pixel FCS; it requires simultaneous FCS measurements on multiple length scales (Fig. 1*d*) [17]. In contrast to GPI-anchored molecules, data from LDL-R anchored mGFP (mGFP-TM, black) requires a two-component fit with a faster short-range and a reduced long-range diffusion coefficient (see S7 Fig. for more details). We have investigated the existence of two-component FCS curves in the case of hop-diffusion in detail using simulations. Fig. 1*e* shows comparison of free diffusion curves (interacting/not interacting with sub-microscopic domains) with the two-component hop-diffusion curve for simulations (see SI for details and S1 Table for a summary of the simulation parameters).

### In intact cells, bimFCS quantifies the different mechanisms causing the reduced diffusion of membrane proteins

Fig. 1*d* (top) shows how bimFCS distinguishes the three forms of diffusion in the cell membrane when the *D*_*app*_ determined for each bin size is plotted as function of binned-pixel size. The *D*_*app*_ of mGFP-GPI (green, empty circles) in PtK2 cells increases with observation area. When the same data is plotted as transit time versus observation size *ω* ^2^, it shows a linear relationship with a positive-axis intercept (*t*_0_) (Fig. 1*d*, bottom). The slope of the linear fit may be used to define the effective diffusion coefficient *D*_*eff*_ = 1/(4*slope). In cells with oxidized membrane cholesterol, the *D*_*app*_ obtained of mGFP-GPI (blue, Δ) was shifted to higher values for all observation sizes (top), and the *t*_0_ was reduced (bottom). In contrast to mGFP-GPI, the *D*_*app*_ obtained from mGFP-TM (black, empty squares) decreases with observation area (top) indicating its interaction with the cytoskeleton.

In the Monte-Carlo simulations of molecules slowed by hop-diffusion the *D*_*app*_ decreases with increasing observation area (Fig. 1*f*, top, black, empty squares). In contrast, for molecules transiently trapped in sub-resolution domains the *D*_*app*_ increases with increasing observation area (green, empty circles). A reduced interaction with the trapping domains results in a shift of *D*_*app*_ to higher values for all observation sizes (blue, empty triangles). Hence, the measured *ω* ^2^-dependence of *D*_*app*_ of mGFP-GPI (Fig. 1*d*, top) indicates that the diffusive behavior of this protein is dominated by transient trapping in sub-resolution domains, and that cholesterol oxidization weakens this trapping [22].

Fig. 1*f* (bottom), shows that *t*_*D*_ versus *ω* ^2^ relationships of the results of the PSF-convoluted simulations are linear, with a negative intercept *t*_0_ for hop-diffusion, a positive *t*_0_ for transient trapping, and a less positive *t*_0_ for weaker trapping. The agreement between the simulation and experimental results shows that bimFCS correctly identifies diffusion slowed by transient trapping in sub-microscopic domains and that the value of the intercept, *t*_0_, may be used to characterize the strength of the molecule’s interaction with the domain. Super-resolution simulations show that all *t*_*D*_ versus *ω* ^2^ relationships bend towards the origin, whereby the position of the bend depends on the domain size. For structures in the cell membrane, however, this regime is not accessible with standard optical microscopy, but may be explored with super-resolution methods [23].

### Comparison of the *t*_0_ and *D*_*eff*_ values obtained from several proteins, under various chemical treatments and in different membrane environments (Fig. 2)

**Fig 2:**
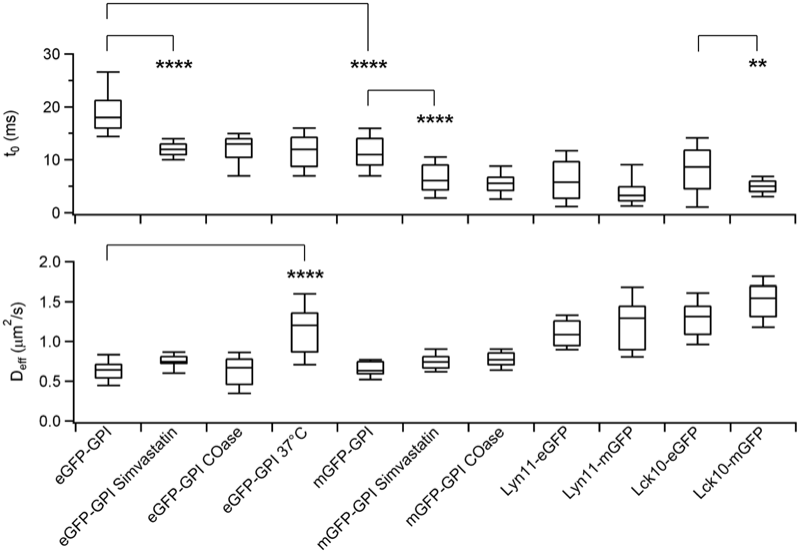
bimFCS technique applied to different membrane proteins under various controlled perturbations. Box and whisker plots of *t*_0_ and *D*_*eff*_ values obtained on mGFP and eGFP tagged outer leaflet (GPI) and inner leaflet (Lyn11 and Lck10) raft markers under various conditions (Number of cells for each case are *N* = 26, 9, 13, 22, 19, 15, 12, 13, 13, 12, 22 consecutively). Cholesterol reduction treatments (Simvastatin and COase) are shown to decrease *t*_0_ significantly (*p*<10^-7^) without effecting *D*_*eff*_ values for both eGFP-GPI and mGFP-GPI. At 37°C eGFP-GPI has a smaller *t*_0_ and a higher *D*_*eff*_ compared to the room temperature (RT, 25°C) controls. All other results were obtained at RT.

On the outer membrane leaflet we found that the GPI anchored enhanced GFP (eGFP-GPI) and mGFP-GPI both exhibit positive *t*_0_ values, which significantly decrease upon membrane cholesterol reduction (with Simvastatin, 100 nM, 24h, *p*<10^-7^) or oxidization (with Cholesterol-oxidase, COase, 1 U/mL, 15 min). Increasing the temperature from 23**°**C to 37**°**C increases the *D*_*eff*_ of eGFP-GPI significantly, while reducing *t*_0_. The results for eGFP-GPI and mGFP-GPI appear mostly independent of the protein expression level (see discussions and S10 Fig.).

The inner membrane leaflet was probed using eGFP or mGFP anchored to the membrane via appended dually acylated amino acid sequences: the N-terminus of Lyn (Lyn11, myristoylated + palmitoylated(24)) or of Lck (Lck10, myr + palm(24) + palm(25)). All of these inner leaflet probes exhibited positive but lower *t*_0_ values than those of GPI-anchored GFP, and had significantly higher *D*_*eff*_. The positive *t*_0_ indicates that these proteins are transiently trapped in sub-resolution domains in the inner leaflet.

Our data show that all eGFP-tagged constructs yield larger *t*_0_ values than their mGFP-tagged counterparts. This difference is statistically significant for the GPI-anchored (*p*<10^-7^) and Lck (*p* = 0.004) constructs, indicating that eGFP-GPI associates with the cholesterol-stabilized domains more strongly than mGFP-GPI does. This stronger interaction may be due the tendency of eGFP to dimerize when attached to a membrane protein [24].

### Continuous bimFCS measurements of GFP-GPI quantify changes in their domain association induced by dimerization

**Fig 3:**
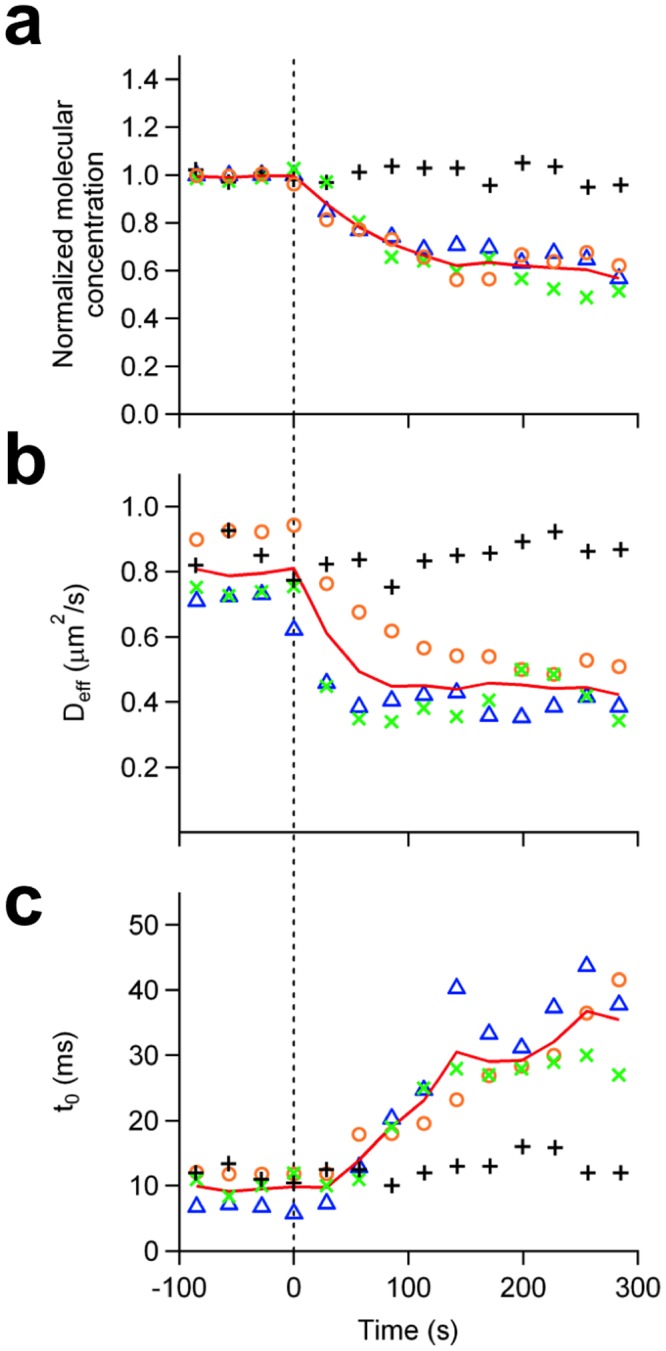
Dynamic changes of the mGFP-GPI interaction with structural domains in single cells upon antibody treatment. Individual responses of three different cells expressing mGFP-GPI to the addition of 2μg/ml monoclonal αGFP antibody (at *t* = 0) are shown (colored markers), with the three-cell average represented in red solid lines. In the case of control cells (black, plus sign), buffer solution without antibodies was added at *t* = 0. (A) Changes of the molecular concentration of diffusers in response to the addition of αGFP antibody. Fluorescent diffuser concentrations are corrected for photo-bleaching (see SI text for details) and normalized to their starting values. (B) Change of the mGFP-GPI effective diffusion coefficient upon αGFP antibody addition. (C) Change of time intercept in response to addition of αGFP antibody.

The molecular concentration, effective diffusion coefficient *D*_*eff*_, and *t*_0_ of mGFP-GPI are highly conserved in the same region on single, unperturbed PtK2 cells (*C*_*norm*_ = 1.00±0.03,*D*_*eff*_ *=* 0.85±0.05 μm^2^, t_0_ = 17±2 ms; control solution added) (Fig. 3). To dimerize the mGFP-GPI molecules a mouse monoclonal anti-GFP antibody (#ab291, Abcam, Cambridge, MA) was added to the cells at 1 μg/ml final concentration while acquiring bimFCS data continuously. The fluorophore density dropped to about half the starting value (61%±2%), confirming effective dimerization of mGFP-GPI (Fig. 3*a*). The *D*_*eff*_ of mGFP-GPI was reduced to about half the initial value, from 0.80±0.01 *μm* ^2^/*s* (averaged over 2 min. before treatment) to 0.44±0.01*μm* ^2^/*s* (averaged from 3 to 5 min. after treatment) (Fig. 3*b*). When dimerizing Liss-Rhod-PE in a supported bilayer using a monoclonal anti-Rhodamine, the *D*_*eff*_ of Liss-Rhod-PE was only reduced by 30% (S8 Fig., *middle*). This indicates that the higher viscous drag of the mGFP-GPI dimers is not sufficient to explain the extent of the decrease in *D*_*eff*_. Hence, the interaction of the dimers with the cholesterol-stabilized domains differs from that of the monomers. The intercept *t*_0_ increases threefold (from 9.6±0.4*ms* to 32±3*ms*) (Fig. 3*c*). This is a larger change than what would be expected from only a viscous drag increase, and indicates that the dimers more strongly associate with the structural domains than do the monomers. Crosslinking of eGFP-GPI with αGFP antibody shows similar results as does mGFP-GPI, with molecular concentration and *D*_*eff*_ both decreasing about 50% and *t*_0_ increasing by threefold, albeit starting at a higher value (S9 Fig.).

### Dimerization of mGFP-GPI influences the interaction of mCherry-GPI with structural domains and vice versa

**Fig 4:**
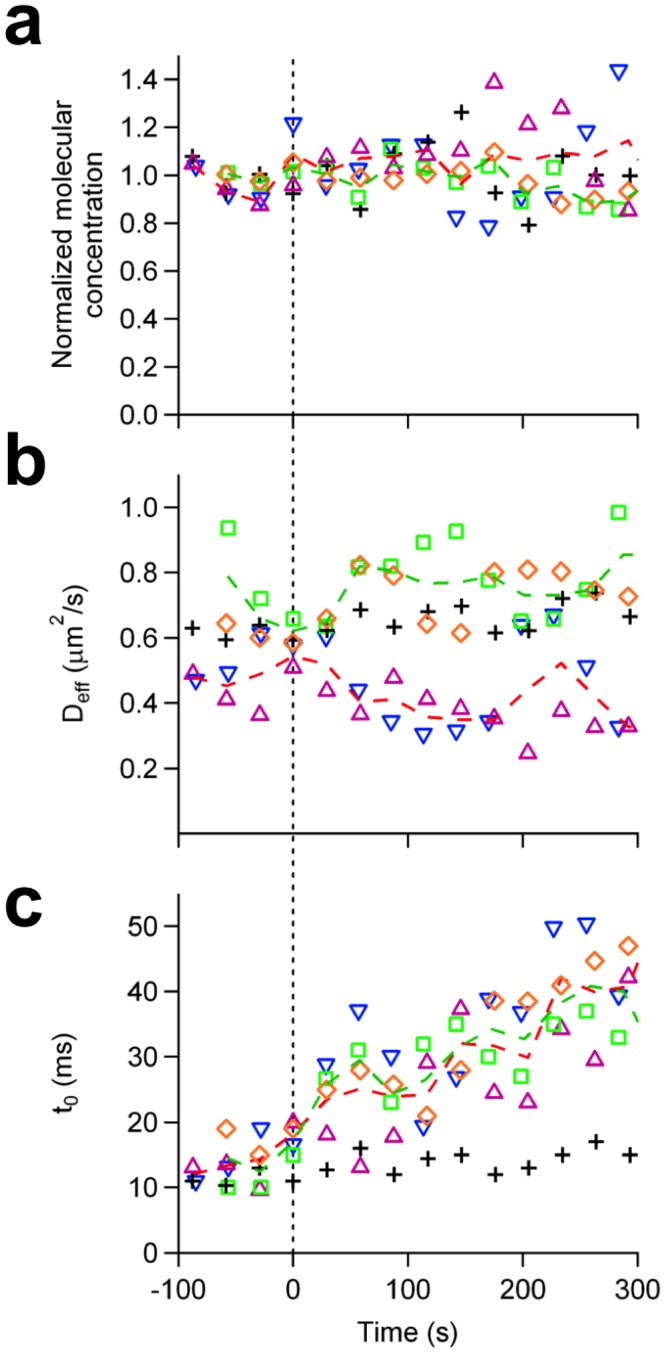
Effect of GPI-protein’s dimerization on cholesterol-stabilized domains quantified by observing another GPI-protein’s domain association simultaneously. (A) Time-course of normalized molecular concentration of mGFP-GPI in response to the addition of monoclonal αRFP antibody which dimerizes the co-expressed mCherry-GPI proteins (diamonds, two individual responses, color-coded, average shown as green dashed line). The bimFCS measurements are repeated for mCherry-GPI upon dimerization of the co-expressed mGFP-GPI by a monoclonal αGFP antibody (triangles, two individual responses, color-coded, average shown as red dashed line). Control trace (black, plus signs) was obtained when the same amount of buffer solution is added at t = 0. All concentration traces are similar to the control trace indicating observed diffusers remain monomeric upon counterpart antibody treatments. (B) Response of the effective diffusion coefficient to antibody treatments. (C) Change of the time intercept in response to antibody treatments.

It is possible; however, that dimerization of GPI-anchored proteins could alter the properties of the structural domains themselves. In order to investigate this possibility, we co-transfected the cells with mGFP-GPI and mCherry-GPI, and took continuous bimFCS data on mGFP-GPI while crosslinking mCherry-GPI molecules with monoclonal anti-RFP antibodies, which do not cross-react with GFP ((#AB-332, ATS, San Diego, CA). The dimerization of mCherry-GPI did not change the molecular density of mGFP-GPI, confirming that the mGFP-GPI molecules remained monomeric (Fig. 4*a*, green, dashed line). Antibody treatment of mCherry-GPI increased the *t*_0_ of mGFP-GPI gradually (from 12.5 ms to 40.0 ms) without significantly changing its *D*_*eff*_ (averaging 0.73 μm^2^/s before the treatment, 0.76 μm^2^/s afterwards) (Fig. 4*b*, *c*, green, dashed line). Similar results were seen when observing mCherry-GPI while dimerizing mGFP-GPI with monoclonal anti-GFP antibody (*t*_0_ is increasing from 12.1 ms to 40.8 ms, and the *D*_*eff*_ is averaging 0.47 μm^2^/s before and 0.41 μm^2^/s after the treatment) (Fig. 4*a*, *b*, *c*, red, dashed line).

Since monoclonal anti-RFP antibody dimerizes mCherry without dimerizing the mGFP, the change of diffusive behavior of mGFP-GPI observed can only be accredited to the alteration of the structural domains with which both mCherry-GPI and mGFP-GPI interact. These results show that dimerizing GPI-anchored proteins modifies the domains in intact living cells.

### Scanning bimFCS measurements on single cells reveal spatial variations across the cell surface, which correlate with the density of α-Actinin

**Fig 5:**
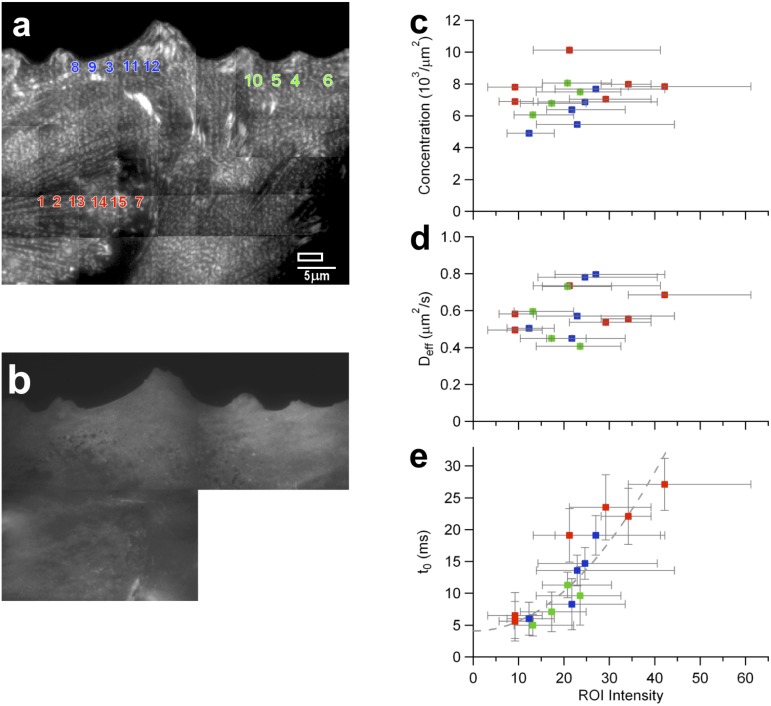
Spatial variations of mGFP-GPI interaction with cholesterol-stabilized domains correlate with α-Actinin density across cell surface. Total-internal reflection fluorescent images of a PtK2 cell expressing α-Actinin-mCherry as a marker for membrane cytoskeleton density (A) and mGFP-GPI as bimFCS probe interacting with membrane nanodomains (B). To cover most of the cell, multiple shifted images were acquired. bimFCS data sets were recorded consecutively from 3 different ROI’s (red, green and blue) and analyzed for 50x20 pixel size sub-ROIs separately (numbered, the size of a sub-ROI is shown above the scale bar). The molecular concentration (C), *D*_*eff*_ (D) and *t*_0_ (E) values obtained from bimFCS analysis of sub-ROIs at different locations within the same cell are plotted against the average, back-ground corrected with α-Actinin-mCherry fluorescence intensity of the respective ROI. Error bars for α-Actinin-mCherry intensity indicate 25 and 75 percentiles of the distribution within each analyzed sub-ROI. Sub-ROIs are numbered according to their α-Actinin-mCherry density.

While the bimFCS parameters are highly reproducible in one ROI of a cell (Fig. 3, control data), we found a much wider spread between cells (Fig. 2) and also across the surface of single cells (S6 Fig.). To understand the origin of the spatial variations within single cells, we correlated bimFCS parameters of mGFP-GPI with the actin membrane cytoskeleton density as recorded by α-Actinin-mCherry fluorescence intensity. BimFCS data were collected simultaneously in different sub-ROIs (50 x 20 pixels) of larger regions (300 x 20 pixels), which were then moved across the surface of *single* PtK2 cells (Fig. 5*a*). The mGFP-GPI molecules are homogenously distributed across the entire cell surface (Fig. 5*b*), as confirmed by the molecular concentration measurement (Fig. 5*c*). The detected spatial variations of the concentrations are within our measurement precision. *D*_*eff*_ varies about 2σ of control measurements, but these variations are uncorrelated to the α-Actinin-mCherry density (Fig. 5*d*). However, the *t*_0_ values correlate positively with αActinin-mCherry density (Fig. 5*e*). Unusually high *t*_0_ values (greater than the 75 percentile of the data obtained across all cells) are found in regions of very high αActinin density (top 25%). Pearson and Spearman coefficients for *t*_0_ vs. ROI intensity are calculated to be *ρ*_*P*_ = 0.85∓0.06 and *ρ*_*S*_ = 0.85∓0.08 (*N* = 4), confirming a strong correlation. The data is fitted well by a quadratic function (χ^2^ = 0.9, a linear fit gives χ^2^ = 1.3) with a significant positive offset in the absence of α-Actinin (*t*_0,*zero actinin*_ *=* 4.1*ms*).

## Discussion

The importance of spatio-temporal control of the distribution of membrane signaling proteins has been well established [25,26]. Yet, only few details of the processes governing the spatial-temporal distribution of these molecules are known, even less is understood about how receptor activation changes this distribution [25,27,28]. A major hurdle has been the lack of experimental techniques, which could quantify the spatial-temporal distribution of receptors during an actual signaling event with sufficient statistics, temporal and spatial resolution on a single cell. Such a method should not interfere with the studied signaling, be compatible with living cells, and be affordable and simple enough for a wide range of users. Our combination of multi-length scale FCS with TIRF imaging provides such a method.

### Resolving membrane protein-domain interactions continuously in single intact cells

One successful approach to study the membrane ultrastructures and the resulting receptor distribution has been diffusion measurement in intact living cells. However, single protein tracking lacks the statistics required to correlate temporal changes in receptor behavior to variations in spatial local structure. FCS analyzing of the fluctuation of many proteins provides the temporal resolution but lacks spatial resolution. Combining TIRF imaging with FCS, and performing TIRF-FCS on multiple length scales simultaneously allows us to quantify the interactions of membrane proteins with the membrane ultrastructure in intact cells without any perturbation except for the introduction of a fluorescent label on the protein. Diffusion of multiple membrane proteins may be analyzed simultaneously using spectrally distinct dyes (dual color bimFCS). Changes in membrane interactions of proteins for different ROIs can be compared simultaneously. This method may be used to study differences between different membrane proteins and cell treatments by averaging the results from a few cells.

Our approach leads us to distinguish and quantify interactions of different membrane proteins with cholesterol stabilized domains, and cytoskeleton fences. We applied the approach to characterize the interaction of various outer- and inner-membrane leaflet proteins with cholesterol-stabilized domains, which historically are sometimes referred to as ‘lipid rafts’[10]. Our experiments show the approach to be sensitive enough to detect difference between inner-leaflet and outer-leaflet lipid anchors and even effects caused by small changes in the ectodomain (eGFP-GPI versus mGFP-GPI). Our antibody treatment results demonstrate how time-traces of bimFCS may be used to study receptor signaling. In this study we focused on the lipid membrane induced ultrastructure. Hence, we refrained from real receptor activation because the resulting cellular signaling may evoke second messengers and cytoskeleton rearrangements, which may send feedback to the cell membrane ultrastructure and over-shadow the lipid membrane effects. In addition, this spatially resolved FCS collects data from a sufficient number of molecules simultaneously. This allows us to deduce information about the local membrane ultrastructure in the observed area of single cells. Spatially resolved dual-color bimFCS can be used to investigate correlations between cholesterol-stabilized lipid rafts and the membrane cytoskeleton on a single cell.

### Practical considerations for applying bimFCS in intact cells

Any diffusing membrane protein or lipid may be characterized by bimFCS as long as it only transiently stalls in clusters or domains and can be fluorescently labeled. As the method is very sensitive to dimerization, even weak changes in protein-protein or protein-lipid interaction are measureable. Using our method, we found a novel monovalent Streptavidin not to perturb the receptor, while an anti-GFP nanobody induced a fraction of dimers [29]. Monomeric GFP, which has been optimized to remain isolated even on a membrane [24], is a useful label for bimFCS, while enhanced GFP (eGFP) causes some dimer formation. GFPs’ photo physical properties are sufficient for bimFCS, but the limited signal-to-noise ratio requires more temporal and spatial averaging. Synthetic fluorophores with higher single molecule brightness and greater photostability than GFP allow shorter exposure times and acquisition periods. Using GFP labeled proteins, we demonstrate that bimFCS data may be gathered continuously from single cells for up to 10 minutes and that *t*_0_ may be determined with sufficient resolution from datasets as short as 30–60 seconds. To obtain reliable diffusion data from FCS, the measurement interval should be 1,000 times longer than the expected diffusion time through the pixel, which for the larger observation sizes may require more than 100 seconds of data [30]. The temporal resolution *τ*_min_ required to measure diffusion coefficients from FCS should be 2/3 of the transit time for a single pixel [31]. GFP-GPI diffuses at room temperature through a diffraction limited area in about 4ms, so we used an exposure time of 1.7ms. The higher single molecule brightness of the Rhodamine labeled lipids used in the supported lipid bilayers (SLB) allowed an exposure time of 0.9ms which was sufficient to resolve the faster diffusion in the SLB correctly (Details in SI).

## Conclusions

### Our data fits best with a *lipid raft model*, which combines dynamic lipid-phase separation with protein induced stabilization and pinning of domains on the cytoskeleton

Two decades ago, *lipid rafts* were proposed to form in the cell membrane by lipid phase-separation and to be stabilized by cholesterol [2,10]. The biological importance of these domains for cell signaling has previously been confirmed. However, there is little consensus about their structure in resting cells or how this changes during signaling. The minimal consensus is that the domains are nanometer-size and signaling proteins interact with them transiently [10]. The models differ in the roles of lipids and proteins in domain formation. Some see the domains’ origins purely in lipid phase separation [2], others combine highly dynamic lipid phase separated domains with pinning to cytoskeleton proteins [32,33], and some propose lipid shells around proteins depending only on the proteins [34].

Our bimFCS results presented here point to a very dynamic and interactive structure that combines elements of several models. Using GPI-anchored fluorescent proteins, we find that their diffusion is reduced by interacting with sub-resolution structures, which may be disrupted by reduction or oxidization of cholesterol. This agrees with many other studies and verifies the existence of cholesterol-stabilized domains in the outer membrane leaflet.

By comparing the strength of this trapping with the density of a cytoskeleton marker across the surface of individual cells, we find that the cytoskeleton increases stability, density or size of domains leading to an increased trapping of GPI. Others previously presented that depolymerizing the membrane cytoskeleton leads to a disappearance of indicators for lipid nanodomains. We find a more differentiated picture—a square dependence on the density of cytoskeleton as may be expected from pinning centers on a surface. Recently, it has been shown in a model system of actin filaments on a supported bilayer with two lipids, that lipid nanodomains of different phases form around pining centers on the filaments[5]. Our data is a first indication that this is also the case in intact cells. Importantly however, our data shows that even in the absence of the cytoskeleton pinning, some weak trapping in the domains exists. Hence, for resting cells our data support a model of dynamic lipid-phase separation driven cholesterol stabilized domains, which are further stabilized by pinning on membrane-cytoskeleton associated proteins.

In addition, the diffusing proteins themselves may serve as nucleation centers for domains [35]. We observe that eGFP-GPI with a weak tendency to form dimers is trapped more strongly in domains than the monomeric GFP-GPI. Similar observations were made recently by Suzuki et al. [36]. To study the influence of receptor dimerization on the membrane domains we dimerize mGFP-GPI with a monoclonal antibody. We find that dimer formation precedes the increased trapping in the domains by tens of seconds. If dimers would purely have a higher retention rate in existing domains, then the change in diffusive behavior should be visible as fast as the drop in molecular concentration, because dimers encounter domains multiple times in one second. The observed lag indicates that the dimers also alter the structural domains (density, stability or size) in a way, which increases trapping. Furthermore, we observe that dimerizing one GPI-anchored protein (mGFP) increases the trapping of another GPI-anchored protein (mCherry) and vice versa. The dimerization affects the other GPI-anchored proteins slower than the dimerized GPI-anchored protein itself. Hence, the antibody addition caused at least two membrane changes. First, dimerization of a protein with some affinity to a domain immediately increases the affinity of that protein to the domain, which will increase their interaction with existing domains. Second, the dimers may provide pinning or nucleation points, which could grow or stabilize an existing domain, or form a new domain around the diffusing dimer. The later would influence other lipid raft proteins. Analyzing data from cells with varying GPI-protein expression levels (from 400 to 8,000 per μm^2^), we find *t*_0_ to be concentration independent and *D*_*eff*_ to be slightly negatively correlated with concentration (S10 Fig.). Apparently, even a 20-fold increase of the expression a monomeric raft protein does not affect the distribution of the raft nanodomains. Interestingly, the measured *t*_0_ values for eGFP-GPI have a larger spread than for mGFP-GPI. They reach from the largest mGFP-GPI value to the lowest antibody dimerized mGFP-GPI measurement. For eGFP-GPI *t*_0_ and *D*_*eff*_ are correlated to each other (S10 Fig.), agreeing with a model in which preformed eGFP-GPI dimers nucleate or stabilize domains [35,36].

In summary, bimFCS provides a novel approach to study spatio-temporal variation in the membrane lipid domains and cytoskeleton structure. Our results support a model explaining cholesterol-stabilized domains as highly dynamic lipid-phase separated domains, which are further stabilized by clustering of diffusing lipid-raft proteins and interactions with the membrane cytoskeleton.

## Supporting Information

S1 File Supplementary methods and protocols. Sample preparation methods for cell culture and supported lipid bilayer.Protocols for BimFCS, such as angle adjustment, temporal requirements, effective beam waist calculation, and bleach correction and power limitations.Details on the Monte-Carlo Simulations and simulation analysis.
(DOCX)

S1 Fig Effective Detection Profile and Equivalent Beam Waist. Color and contour plot of square-detection profile as the convolution of square pixel with 2D Gaussian PSF. Dark red dashed circle represents averaged out circle of 1/*e* ^2^ threshold (brown) with radius giving the detection waist, *ω*.
(TIF)

S2 Fig Data Analysis Software Flow Diagram. A flow diagram of the data analysis implemented in IgorPro (Wavemetrics).
(TIF)

S3 Fig Effect of Bleach Correction. **(*A*)** The need for and effects of bleach correction are explained on data taken from a lipid bilayer in which 0.025% of the PE lipids were labeled with Rhodamine. (***Top***) The spatially averaged frame intensities before (*left*) and after (*right*) bleach correction were plotted over time. (***Middle***) Relative fluorescence fluctuations in a 3×3 binned area before (*left*) and after (*right*) bleach correction. Relative fluorescence signal was calculated by subtracting the time-average signal and then dividing the differential signal by the time average value. (***Bottom***) A comparison of the resultant FCS curves shows that: without bleach correction, photobleaching (in the timescale of seconds) was the dominant decay mechanism (*left*); the shorter decay time due to diffusion (in tens of ms) only becomes apparent after bleach correction was performed (*right*). (***B***) Bleaching only reduces the number of diffusing molecules and does not affect the single molecule brightness. Data presented here was taken from a lipid bilayer in which 0.05% of the PE lipids were labeled with Rhodamine. For a set of long time-course data over 4 minutes, concatenated, exponential fit of the average frame intensities gives a time constant *τ*_*exp*_ = 54.2±0.2 *s* (*left*). First 20 seconds of the acquisition (black) is masked, as done for all data. Molecular concentrations calculated from the amplitudes of AC curves, through time-course bimFCS analysis (averaging every 32Kframes), shows an exponential decay with *τ*_*exp*_ = 60±10 *s* (*right*). The single molecule brightness of each Rhodamine is plotted versus time in the *inset*. (***C***) Excitation Laser Power Dependence of bimFCS Data. Transit time values calculated from FCS curves obtained at small and large bin sizes (red, filled, *ω* ^2^ = 0.0717 *μm* ^2^; blue, open, *ω* ^2^ = 0.2216 *μm* ^2^) as a function of laser power at the objective lens. Data is acquired from mGFP-GPI diffusing in PtK2 cell membrane.
(TIF)

S4 Fig Camera-FCS Curves & Fits for Liss-Rhod Diffusion in Lipid Bilayers. FCS curves obtained at two different bin sizes (red, filled, *ω* ^2^ = 0.0688 *μm* ^2^; blue, open, *ω* ^2^ = 0.1116 *μm* ^2^) from supported lipid bilayers (SLB) with 2 different LissRhod PE to DOPC ratios (circles, 1:10000; triangles, 1:30000) showing that the correlation function amplitude decreases with increasing fluorophore density and observation area. Fractional Residues (residue/data) for two different bin sizes from two different mixture ratios data is shown on top of the graph.
Systematic error between fit and data for small bin size of low concentration data marks the limit of FCS technique in terms of number of diffusers.
(TIF)

S5 Fig Concentration of Diffusers Calculated for Experimental & Simulation Data. Molecular concentration data is plotted versus observation area *ω* ^2^ for LissRhod PE in a SLB (black, filled triangles, *N* = 9) and overlaid with results from two (super resolution & microscope optics) Monte-Carlo Simulations. The experimental results (black, filled triangles, right axis) were obtained from a SLB with LissRhod PE:DOPC ratio of 1:10000. Number of fluorescent diffusers per unit area shows no dependence on pixel size. MC simulation of known molecular density (blue, dashed line: time average of diffuser density as determined by total number of molecules divided by total simulation area. Grey lines: ± standard deviation σ) were performed and analyzed with (red, empty squares, left axis) and without (green, empty circles, left axis) convolution with PSF. The molecular densities calculated from the resulting FCS curves of simulations are within 1σ with respect to the expected value.
(TIF)

S6 Fig Spatially Resolved bimFCS Data over an ROI from Single Cell Membrane. (a) TIRF camera images of the GPI anchored mGFP molecules in a PtK2 cell overlapped with 5 m shifts of the stage. Increasing wavelength rainbow colors indicate sequence of data acquisition for each ROI (100x20 pixels). (b) Time intercept values from the linear fit of the FCS law plots of bimFCS data, averaged over each ROI. (c) Effective diffusion coefficient from the linear fit of FCS law plots of bimFCS data.
(TIF)

S7 Fig Effect of Jasplakinolide Treatment on Diffusion of Transmembrane Protein. Normalized FCS curves (*ω* ^2^ = 0.087 *μm* ^2^) obtained from mGFP-TM (empty squares), and of mGFP-TM after incubating the cell with the actin filament disrupting drug Jasplakinolide treatment (plus signs), in PtK2 cells at 37°C. Data is fitted with two diffusion component square-pinhole FCS function. The original FCS curve clearly shows two diffusion coefficients (*χ* ^2^_*doublefit*_/*χ* ^2^_*singlefit*_ = 0.039). After Jasplakinolide, the shape of FCS curve approximates that of free Brownian motion (*χ* ^2^_*doublefit*_/*χ* ^2^_*singlefit*_ = 0.109), confirming that disruption of actin filaments removes the slower long-distance diffusion of the transmembrane protein.
(TIF)

S8 Fig Dimerization of Liss-Rhod-PE with Monoclonal αRhodamine Antibody. Changes of molecular concentration (*top*), effective diffusion coefficient (*middle*), and time axis intercept (*bottom*) of Liss-Rhod-PE diffusing in a supported lipid bilayer in response to the addition of αRhodamine antibody (2μg/ml) are shown. Time courses obtained from two separate experiments are shown (red plus sign and blue empty triangle), with the average between the two plotted in black dashed line. The normalized diffuser concentration dropped by around 50%. The *t*_0_ value stayed around 0 throughout the time course. The effective diffusion coefficient of Liss-Rhod-PE dropped to about 70% of its starting value upon αRhodamine antibody treatment, compared to 50% in the case of mGFP-GPI dimerization.
(TIF)

S9 Fig Dynamic Changes of the eGFP-GPI Interaction with Nanodomains in Single Cell upon Monoclonal αGFP Antibody Treatment. Changes of molecular concentration (*top*), effective diffusion coefficient (*middle*), and time axis intercept (*bottom*) of eGFP-GPI in response to the addition of αGFP antibody are shown (red, filled circles) together with the respective control traces (black, empty squares). Monoclonal αGFP antibody was added at t = 0 to a final concentration of 2μg/ml, while the same amount of buffer without antibodies was added to the control cell. Similar to the results from mGFP-GPI, the molecular concentration of eGFP-GPI dropped by 50%. This confirms effective dimerization induced by antibody cross linking. The effective diffusion coefficient of eGFP-GPI dropped to about 60% of its starting value, and the *t*_0_value increased by three fold.
(TIF)

S10 Fig Concentration Dependence of *D*_*eff*_ and *t*_0_ Obtained from mGFP-GPI and eGFP-GPI, and Their Correlation with Each Other. Effective diffusion coefficient (top) and time axis intercept (middle) obtained from mGFP-GPI (red, empty circles) and eGFP-GPI (blue, plus signs) are plotted as a function of measured molecular concentration. A slight negative correlation is found between D_eff_ and molecular concentration for both mGFP-GPI (Pearson coefficient *ρ*_*p*_ = -0.52) and eGFP-GPI (*ρ*_*p*_ = -0.44), while *t*_0_ is not dependent on molecular density. The correlation between *t*_0_ and D_eff_ is represented by plotting the slope of the linear fit, which is mathematically equal to 1/4D_eff_, versus *t*_0_ for each measurement (*C*). The Pearson coefficient between slope and *t*_0_ is 0.13 for mGFP-GPI and 0.44 for eGFP-GPI. bimFCS can be successfully performed on GFP labeled proteins expressing between 400 and 8,000 molecules/m^2^, more than an order of magnitude. Below this, the fluctuations are too small and rare compared to background and above this concentration, the fluctuations are too small of a fraction of the total signal. Using a brighter fluorophore, one can reach low concentrations, down to 50 molecules/m^2^ for Rhodamine. Over this range, we find little correlation between concentration and of *D*_*eff*_ or *t*_0_ or indicating that the heterologously protein expression does not affect the cell membrane ultrastructure.
(TIF)

S1 Table Description of Simulation Parameters. Parameters used in the Monte-Carlo Simulation of bimFCS of tracers diffusing on a regular grid with membrane fences and local traps.
(DOCX)
