# Supplementary material for: Effect of Receptor Dimerization on Membrane Lipid Raft Structure Continuously Quantified on Single Cells by Camera Based Fluorescence Correlation Spectroscopy
Source: PLoS One. 2015 Mar 26;10(3):e0121777. doi: 10.1371/journal.pone.0121777 (PMC4374828; doi:10.1371/journal.pone.0121777)
Supplement: S1 File — Sample preparation methods for cell culture and supported lipid bilayer.Protocols for BimFCS, such as angle adjustment, temporal requirements, effective beam waist calculation, and bleach correction and power limitations.Details on the Monte-Carlo Simulations and simulation analysis. (DOCX) [file pone.0121777.s001.docx]

**SUPPLEMENTARY INFORMATION**

**Experimental Methods**

**Cell Culture**

PtK2 (Male Rat Kangaroo Kidney Epithelial Cells) cells were cultured in Eagle’s minimum essential medium supplemented with 10% fetal bovine serum, 1% penicillin-streptomycin, 1% non-essential amino acid and 1% sodium pyruvate (Invitrogen) at 37˚C under 5% CO2. For imaging, the cells were plated sparsely on 18-mm glass coverslips coated with 0.05% poly-L-lysine hydrobromide (Sigma). Transfection of PtK2 cells were performed 24 h after plating using Lipofectamine 2000 (Invitrogen).

Simvastatin (Sigma Aldrich) treatments were performed at 100nM concentration in the incubator (37˚C and 5% CO_2_) overnight before bimFCS analysis. Treatments of cells with Cholesterol Oxidase (COase from Streptomyces, Sigma Aldrich) were done at 1U/ml concentration at 37˚C for 15min. Treatments of cells with Jasplakinolide (Cayman Chemical) were done at 6μM concentration at 37˚C for 20mins. The cells were subsequently washed and imaged in physiological salt solution (PSS, ingredients in mM: CaCl_2_ 2, NaCl 151, MgCl_2_ 1, KCl 5, HEPES 10, Glucose 10 ph 7.3). All bimFCS experiments on live cells were performed in PSS buffer. The mCherry-GPI and eGFP-GPI plasmids were gifts of G. Baron (NIAID/NIH) and P. Keller (MPI-CBG, Dresden), respectively. We derived mGFP-GPI by mutating A206K in eGFP-GPI. The αActinin-mCherry construct was generously provided by T. Reno (UCSD) and R. Horwitz (UVA). We received mGFP-TM construct from A. Kusumi. For dimerization of mGFP-GPI or eGFP-GPI, 2μg of monoclonal anti-GFP antibodies (#ab291, Abcam, Cambridge, MA) was pre-diluted in 70μl of PSS. The resulting solution was then added dropwise into the imaging chamber containing 1ml PSS (resulting in a 2μg/ml final concentration) as bimFCS data was being continuously recorded. For dimerization of mCherry-GPI, monoclonal anti-RFP antibody (#ab332, ATS, San Diego, CA) was used at the same concentration with the same procedure.

**Supported Lipid Bilayer**

1-palmitoyl-2-oleoyl-sn-glycero-3-phosphocholine (POPC), and 1,2-dioleoyl-sn-glycero-3-phosphoethanolamine-N-(lissamine rhodamine B sulfonyl) (Liss Rhod PE) were purchased from Avanti. The coverslips were first treated with Piranha solution (3:1 concentrated Sulfuric acid to 30% hydrogen peroxide solution) at 65 ˚C for 30 to 60 min then rinsed with 18MΩ water. They were stored in air. Two mixtures (2.5% dye-labeled and un-labeled) of lipids in chloroform were dried with a stream of nitrogen and placed under vacuum for 1 hour. Lipids then were hydrated in PBS (1 mg lipids / 1 ml PBS). The resulting vesicles underwent five freeze (liquid nitrogen) – thaw (37 ˚C water bath) cycles before being extruded a minimum of 20 times through polycarbonate membranes with 100 nm diameter pores (Avanti Mini-Extruder). Vesicle solution containing 2.5% dye-labeled lipids was mixed with vesicle solution of un-labeled lipids at 1:100 ratio and then sandwiched between a coverslip and a glass microscope slide for 1 to 2 min. Bilayers were formed by vesicle fusion on the glass coverslip surfaces.

**BimFCS Technique**

**TIR Angle Adjustment**

Two different size fluorescent polystyrene beads (1 and 0.1 μM) in salt solution on coverglass are used to optimize and standardize the evanescent field by adjusting the total internal reflection (TIR) angle. The brightness of smaller size beads are set to be maximum to the extent that the ratio of larger size beads’ brightness to the smaller is lowest. 2D Gaussian fit of TIRF images from fluorescent beads with sizes smaller than the diffraction limit are used to calculate the point spread function (PSF) of the optical setup for the relevant filter set.

**Temporal Requirements for FCS Data Acquisition**

To obtain reliable diffusion measurement from FCS data, the data has to be recorded for a sufficiently long time period, averaging over multiple events, and with a sufficiently high temporal resolution.

For each static measurement, 90s of data were recorded, because determining the time constant of a processing using autocorrelation curves requires the measurement interval to be about 1,000 times longer than the expected time constant of this process [1]. Time courses covering dynamic changes caused by external perturbation were obtained by continuous data acquisitions for 360 s while the reagent to be studied was added after the first 60 s.

The temporal resolution of an EMCDD is sufficient because the typical diffusion coefficient of membrane proteins is only in the order of 1▪10^-12^ m^2^/s. Tcherniak et al. [2] showed that the temporal resolution *τ*_min_ required to recover reproducible diffusion constants in FCS should not exceed 2/3 the value of the characteristic decay time. The transit time for a single pixel is about 4ms, so the temporal resolution has to be smaller than 2.67ms. In our study, the temporal resolution was 1.7ms, although we have also acquired data with 0.7ms. To achieve these read-out times, a rectangular-shaped region of interest (ROI) of 20 rows × 200 pixels is chosen around 3μm from the edge of cell. The finite measurement time of 1.7ms causes a localization uncertainty, which for a membrane protein with a diffusion coefficient D = 1 μm^2^/s corresponds to $4Dt=\left\langle r^{2} \right\rangle\sim\left( 80nm \right)^{2}$. This is negligible compared to the many-fold larger detection area (see next segment).

**Effective beam waist calculation**

In traditional confocal FCS experiments the effective excitation spot size, $\omega^{2}$, is determined by the waist $\omega$ of the focused Gaussian laser beam. For bimFCS, $\omega^{2}$ corresponds to the size of the detection profile, $L_{xy}=L_{x}(x)L_{y}(y)$, which is given by the image of the square pinhole convoluted with the PSF in 2D. This is mathematically expressed ([20](#_ENREF_20)) in one axis as

$L_{x}(x)=\frac{1}{a}\int_{0}^{a} PSF(x-x_{0})dx_{0}=\frac{1}{2a}\left(erf(\frac{a-x}{2^{\frac{1}{2}}\sigma})+erf(\frac{x}{2^{\frac{1}{2}}\sigma})\right)$, (1)

with the PSF (1/√e waist of σ) approximated by a Gaussian. For each bin, the distance from the center of the binned pixel to the position where the detection efficiency drops to $1/e^{2}$ of the central value (in parallel with the definition of beam waist for Gaussian beam) has been computed and averaged with angular step size of 0.001 radians to obtain the corresponding ω value, which is then used to calculate $t_{D}$ value for this particular bin (See Figure S1). Validity of effective beam waist calculation for PSF is tested with FCS law overlays of Kinetic Monte-Carlo simulations, for diffraction limited and super-resolution cases (See “Simultaneous multi-length scale diffusion measurements by bimFCS” in main text.).

**Bleach correction and choosing the correct excitation power**

Bleaching in TIRF FCS may happen in two areas: *First*, GFP could bleach while still diffusing through the observation area used to compute the autocorrelation curve. If this happens, then the correlation curve would be compressed and the calculated diffusion times would fall too short due to missing long transit times. This effect is observable for high excitation powers and large pixel bins (see Figure S3 C). The transit times for small pixel bins are 1 – 20 milliseconds, but reach hundreds of milliseconds for larger pixel bins. This makes bleaching ten times more likely for large bin sizes. However, all presented data is taken with sufficiently low excitation powers and in small ROIs that this doesn’t happen. This form of bleaching is observable in common confocal FCS in an unlimited reservoir of molecules, and effects the estimated diffusion time and concentration. In a limited reservoir, such as the cell membrane, bleaching also depletes the reservoir of GFPs. However, in TIRF-FCS, where the illumination area is larger than each of the ROI (pixel bins) used for FCS calculation, bleaching also occurs outside this ROI. While the ROI used for each FCS curve is at most 0.25µm^2^, we record video data from a much larger area (between 20x100 and 40x400 pixels, corresponding to 8.2um^2^ and 65.5µm^2^) to allow for spatial analysis or averaging. The TIRF excitation spot covers a circle of 200 pixel radius, corresponding to 515µm^2^ which is 2000x larger than the maximal ROI used for FCS calculations. Hence the bleaching outside the ROI (pixel bin) used for FCS is also 2000x larger than inside the pixel bin. This bleaching only reduces the number of GFP molecules, does not affect the FCS data, and can be corrected.

To correct for this bleaching effect, once the raw bimFCS data from each pixel of camera, a stack of tiff images, is loaded into a 3-D intensity matrix, the average intensity of each frame (F_ave_) is calculated and the resultant F_ave_ (t) is fitted with an exponential decay function. (For a detailed flow-chart of bimFCS data analysis see S2 Fig.)

The bleach corrected intensity value of each individual pixel is then calculated as:

$\left. F_{pixel,corrbleach} \right|_{t=t}=\left( \left. F_{pixel} \right|_{t=t}-\left. F_{average} \right|_{t=t} \right)+\left. F_{ave,expfit} \right|_{t=0}$ (2)

This allows the correction for the effects of photobleaching, laser fluctuations and any mechanism that would result in global change of signal intensity. An example of the effects of bleach correction is shown in S3A Fig., side by side.

Hence, for bimFCS, unlike confocal FCS, bleaching occurs only outside the ROI used for FCS and only affects the available number of molecules, which is also visible in the slowly decreasing variance in S3A Fig., middle. It can be shown that this decrease in variance is purely a result of decrease number of molecules, and does not affect the FCS result. As the number of visible molecules decreases, the variance decreases, but the brightness per molecule remains the same. The brightness per molecule calculated from the ratio between average frame intensity and the number of molecules shows a constant trend with 7.4% st.dev. spread about the average (S3B Fig. *right, inset*). Matching time constants for exponential decays of average intensity and concentration of molecules due to bleaching, indicates decreasing variance of corrected intensity data does not inflict FCS diffusion measurements.

Actually calculating the number of molecules provides a good control of the method: (i) across the binning sizes we obtain the same concentration, (ii) in a sample with known concentration of traces (lipid bilayer and simulations) we recover the correct value, and (iii) over time we record exponential decay. This lets us to deduce information about biochemically induced concentration changes, such as dimerization, with time-course data, once corrected exponentially.

Using very low excitation power would avoid photo-bleaching, but if the fluorescence brightness per diffusing molecule becomes to low compared to other noise sources, the FCS curves become to noisy to obtain reliable diffusion estimates. This results in an over-estimation of the transit time for diffusers. We find that this occur excitation powers smaller than 300 W/m^2^ for GFP, or when the molecule brightness calculated from the FCS data drops below ~300 cpsm .

**Experimental Results**

**Concentration of Fluorescent Molecules**

The amplitude of the FCS curve should be inversely proportional to the number of molecules within the observation area (Eq. 2). bimFCS quantified correctly for each particular bin size; the amplitude of the FCS curve scales inversely with fluorescent lipid concentration (threefold lower from the bilayer which has threefold more fluorescent lipids), and that the amplitude scales inversely with observation area when the fluorophore concentration is constant (S4 Fig.). Taken from the same bilayer, normalized FCS curves of different super-pixels show the autocorrelation time increases with increasing pixel size, as expected for a diffusion-dominated process (Fig. 1*a*). The fluorophore density (number of fluorescent molecules per unit area), is found to be independent of observation area as expected (S5 Fig.).

**Indirect Observation of the Effect of Jasplakinolide Treatment on Cytoskeleton**

In the manuscript text we describe that GFP anchored to the membrane by a transmembrane domain (mGFP-TM) experiences two-component (hop) diffusion. To test the interactions with the membrane cytoskeleton slows TM protein’s long-range diffusion, cells transiently expressing mGFP-TM were treated with Jasplakinolide –a chemical drug claimed to disrupt actin filaments-. S7 Fig. displays before and after treatment FCS curves for mGFP-TM. The original FCS curve clearly shows two diffusion coefficients (ratio χ²_doublefit_ / χ²_singlefit_ = 0.039). After Jasplakinolide, the shape of FCS curve approximates that of free Brownian motion (ratio χ²_doublefit_ / χ²_singlefit_ = 0.109), confirming that disruption of actin filaments removes the slower long-distance diffusion of the transmembrane protein.

**Simulations**

**Simulation Details**

Diffusion simulations were performed on a 2 x 2 μm^2^ square lattice with 1 nm lattice spacing, keeping 100 ± 20 molecules within the observation area at any time. To achieve this, a new molecule enters from a random edge position within the simulation area at a prior or later random time as one diffuses out. All pseudorandom numbers were generated using the default random number generator of MATLAB R2009b seeded with the real computer time. The simulations were carried out with 0.4μs (for bilayer free diffusion) temporal and 1nm spatial resolution and then down-sampled to 0.4 ms and 4 nm, respectively.

To simulate the diffusion of molecules transiently trapped in cholesterol enriched nanodomains in the cell membrane, square-sized nano-domains with edge length $l$ were introduced, and arranged in a regular square pattern occupying a defined area fraction, $d$. Diffusing molecules enter these domains freely ($P_{in}=1$) and become temporarily trapped ($P_{out}<1$). Inside the domains the diffusion of the molecules is reduced by $D_{rel}=\frac{D^{in}}{D^{out}}$ (simulated by allowing molecules to remain in place with a certain probability instead of moving to a neighboring position for the next MCS step). The diffusion coefficient outside was chosen to match our experimental DiI results ($D^{out}$=1.30 μm^2^/sec in PtK2 cells, data not shown).

The cytoskeleton meshwork under the cell membrane is modeled as a square-grid of barriers with spacing $a$. Molecules may hop over these barriers with a hopping probability $P$. **S1 Table** summarizes the input parameters for all simulations.

**Simulation Analysis**

To be able to compare the MCS with the experiments, detection of the MCS data with a virtual microscope is simulated. The intensity matrices $IM\left( x,y \right)$ are calculated by convoluting the matrix containing the positions of the simulated molecules, $P\left( x^{'},y^{'} \right)$, with a 2D Gaussian PSF (σ = 108.8 nm):

$IM\left( x,y \right)=PSF\left( x,y,x^{'},y^{'} \right)\bigotimes P\left( x^{'},y^{'} \right)=\frac{1}{\sqrt{2\pi\sigma}}e^{-\frac{\left( x-x^{'} \right)^{2}+\left( y-y^{'} \right)^{2}}{2\sigma^{2}}} \bigotimes P(x^{'},y^{'})$ , (3)

A camera with 64nm x 64nm pixel size records intensities in a 25 x 25 pixel ROI centered within the simulation area, generating a camera frame. The simulation area was chosen to extend 400 nm past the edge of the ROI in each direction to nullify edge effects during convolution of point spread functions. As a control, we also analyzed the simulations as super resolution microscopy (SR) taking each diffuser as a point light source without spatial spreading.

The camera frames are analyzed identically to experimental data (Fig. 1*e-f*). Vertical error bars of simulation output data are determined by the min-max spread of multiple simulation runs with the same conditions. Simulations of various lengths (corresponding to 40 sec – 6 min in real time duration) have been run to compare fidelity of statistics.

**S1 Table** lists the output parameters of the simulations: the partition α of molecules into domains, the confinement strength $S_{conf}$ and from the FCS diffusion law analysis the effective diffusion coefficient $D_{eff}$ and time intercept $t_{0}$. The latter two can be compared to the experimental values.

**S1 Table. Description of Simulation Parameters.**

| **Parameter description** | | | **Symbol** | | **Value / Range of values / Calculation** |
| --- | --- | --- | --- | --- | --- |
| *Monte Carlo parameters* | | | |  |  |
|  | Lattice step size | | |  | $1nm$ |
|  | Simulation box | | |  | $2\mu m\times2\mu m$ |
|  | Observation area | | |  | $1.6\mu m\times1.6\mu m$ |
|  | Total simulated time | | |  | $>40 sec$ |
|  | Number of molecules in frame for FCS | | |  | $100\pm20$ |
| *Parameters to match simulation to experimental conditions* | | | | | |
|  | Free diffusion coefficient | | | $D^{out}$ | in supported lipid bilayer $2.5{{\mu m}^{2}}/{sec}$;  on cells $1.3{{\mu m}^{2}}/{sec}$ |
|  | 2D Gaussian width of PSF | | | $\sigma$ | $108.8nm$ |
|  | Camera pixel size | | |  | $64 nm$ |
|  | Effective waist of detection spot | | | $\omega$ |  |
|  | Camera frame time | | |  | 1.5ms; or 1000 MCS steps |
|  | Number of molecules in each frame | | |  | $100\pm20$ |
| *Specific parameters for nano-domains* | | | |  |  |
|  | Edge length of domains | | | $l$ | $20-75 nm$ |
|  | Fraction of area covered by domains | | | $d$ | $0.1-0.3$ |
|  | Relative diffusion coefficient inside domains | | | $D_{rel}$ | $0.1-1$; ${D^{in}}/{D^{out}}$ |
|  | Probability of entering a domain | | | $P_{in}$ | $1$ |
|  | Probability of exiting a domain | | | $P_{out}$ | $0.01-0.04, 0.1-0.2$ |
| *Specific parameters for membrane fences* | | | |  |  |
|  | | Spacing of square-grid fences | | $a$ | $50-500 nm$ |
|  | | Hopping over probability for molecules | | $P$ | $0.01-0.1$ |
| *Simulation observables* | | | |  |  |
|  | Partition of molecules into domains | | | $\alpha$ | (molecules inside domains) / total molecules |
|  | Confinement strength | | | $S_{conf}$ |  |
|  | Effective diffusion coefficient | | | $D_{eff}$ | $1/{(4*(slope of t_{D} versus \omega^{2}plot))}$ |
|  | Time-axis intercept of the FCS diffusion law | | | $t_{0}$ | y-intercept |
