# Supplementary figures and images for: Effect of Receptor Dimerization on Membrane Lipid Raft Structure Continuously Quantified on Single Cells by Camera Based Fluorescence Correlation Spectroscopy
Source: PLoS One. 2015 Mar 26;10(3):e0121777. doi: 10.1371/journal.pone.0121777 (PMC4374828; doi:10.1371/journal.pone.0121777)

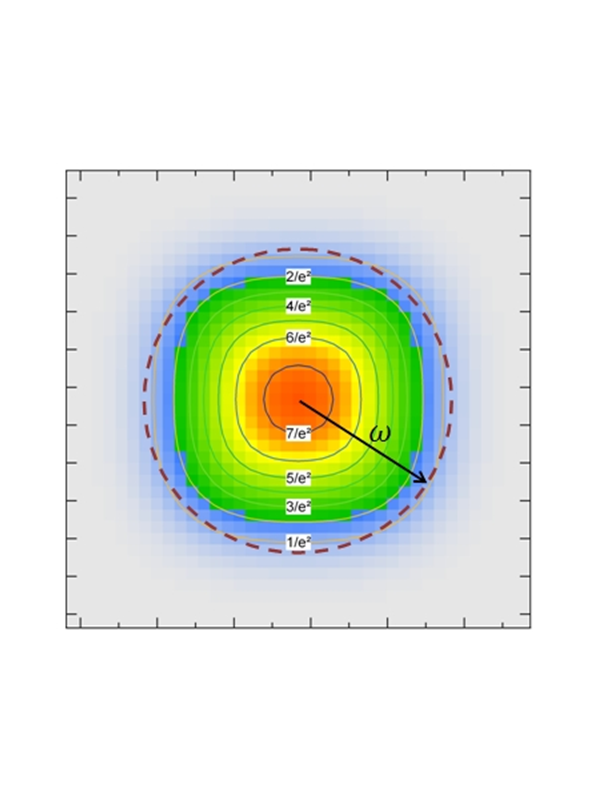

Supplement: S1 Fig — Color and contour plot of square-detection profile as the convolution of square pixel with 2D Gaussian PSF. Dark red dashed circle represents averaged out circle of 1/e 2 threshold (brown) with radius giving the detection waist, ω. (TIF) [file pone.0121777.s002.tif]

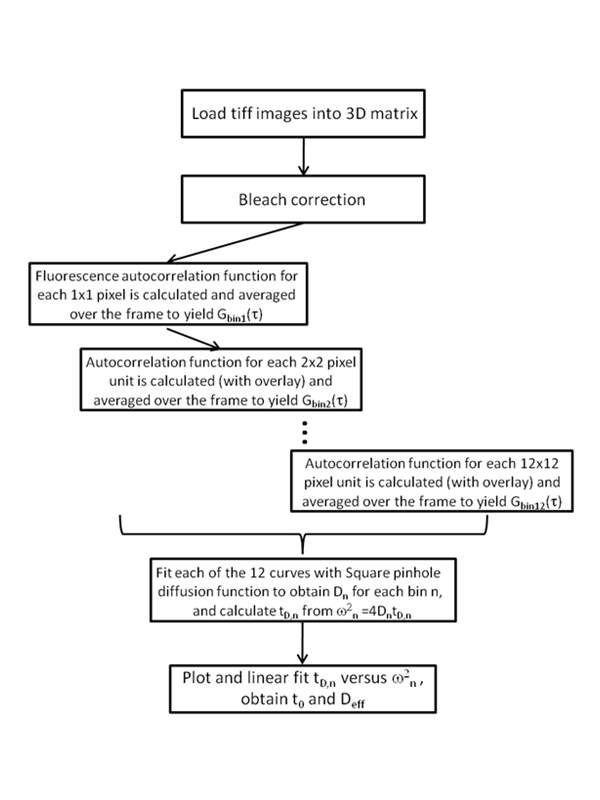

Supplement: S2 Fig — A flow diagram of the data analysis implemented in IgorPro (Wavemetrics). (TIF) [file pone.0121777.s003.tif]

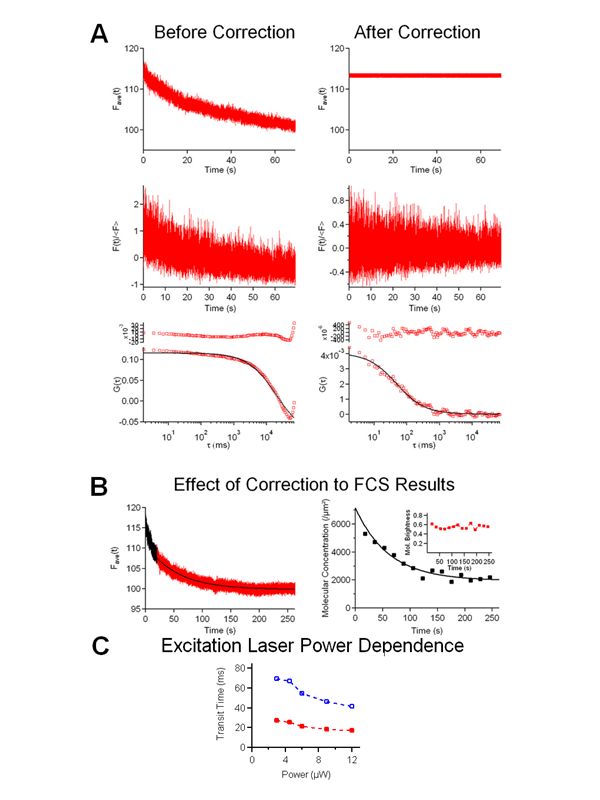

Supplement: S3 Fig — (A) The need for and effects of bleach correction are explained on data taken from a lipid bilayer in which 0.025% of the PE lipids were labeled with Rhodamine. (Top) The spatially averaged frame intensities before (left) and after (right) bleach correction were plotted over time. (Middle) Relative fluorescence fluctuations in a 3×3 binned area before (left) and after (right) bleach correction. Relative fluorescence signal was calculated by subtracting the time-average signal and then dividing the differential signal by the time average value. (Bottom) A comparison of the resultant FCS curves shows that: without bleach correction, photobleaching (in the timescale of seconds) was the dominant decay mechanism (left); the shorter decay time due to diffusion (in tens of ms) only becomes apparent after bleach correction was performed (right). (B) Bleaching only reduces the number of diffusing molecules and does not affect the single molecule brightness. Data presented here was taken from a lipid bilayer in which 0.05% of the PE lipids were labeled with Rhodamine. For a set of long time-course data over 4 minutes, concatenated, exponential fit of the average frame intensities gives a time constant τ exp = 54.2±0.2 s (left). First 20 seconds of the acquisition (black) is masked, as done for all data. Molecular concentrations calculated from the amplitudes of AC curves, through time-course bimFCS analysis (averaging every 32Kframes), shows an exponential decay with τ exp = 60±10 s (right). The single molecule brightness of each Rhodamine is plotted versus time in the inset. (C) Excitation Laser Power Dependence of bimFCS Data. Transit time values calculated from FCS curves obtained at small and large bin sizes (red, filled, ω 2 = 0.0717 μm 2; blue, open, ω 2 = 0.2216 μm 2) as a function of laser power at the objective lens. Data is acquired from mGFP-GPI diffusing in PtK2 cell membrane. (TIF) [file pone.0121777.s004.tif]

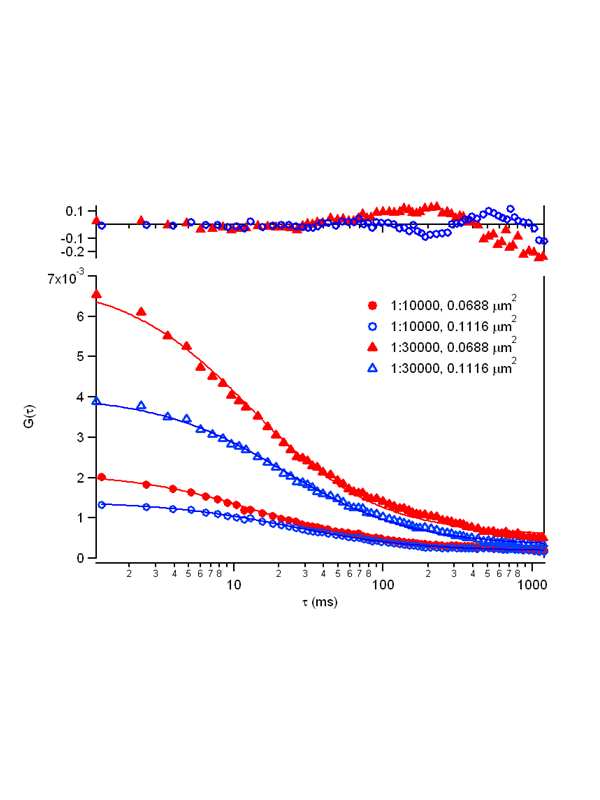

Supplement: S4 Fig — FCS curves obtained at two different bin sizes (red, filled, ω 2 = 0.0688 μm 2; blue, open, ω 2 = 0.1116 μm 2) from supported lipid bilayers (SLB) with 2 different LissRhod PE to DOPC ratios (circles, 1:10000; triangles, 1:30000) showing that the correlation function amplitude decreases with increasing fluorophore density and observation area. Fractional Residues (residue/data) for two different bin sizes from two different mixture ratios data is shown on top of the graph. Systematic error between fit and data for small bin size of low concentration data marks the limit of FCS technique in terms of number of diffusers. (TIF) [file pone.0121777.s005.tif]

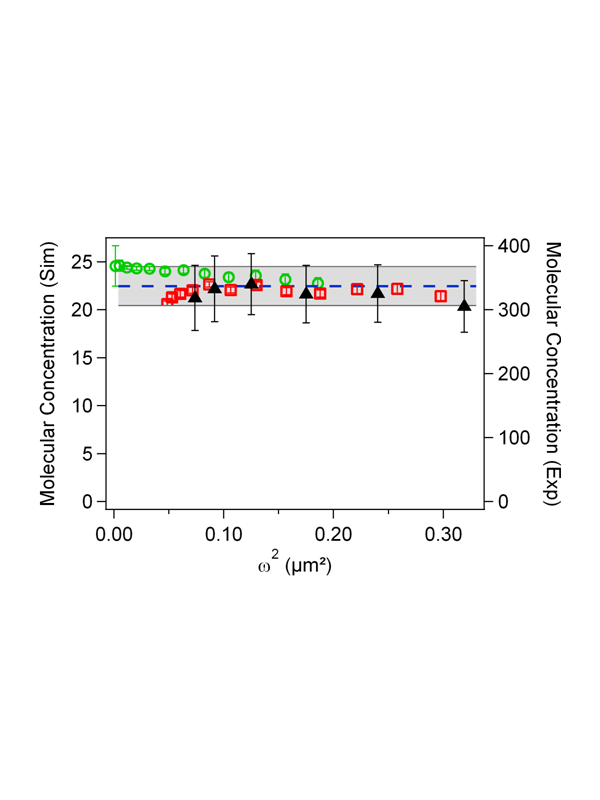

Supplement: S5 Fig — Molecular concentration data is plotted versus observation area ω 2 for LissRhod PE in a SLB (black, filled triangles, N = 9) and overlaid with results from two (super resolution & microscope optics) Monte-Carlo Simulations. The experimental results (black, filled triangles, right axis) were obtained from a SLB with LissRhod PE:DOPC ratio of 1:10000. Number of fluorescent diffusers per unit area shows no dependence on pixel size. MC simulation of known molecular density (blue, dashed line: time average of diffuser density as determined by total number of molecules divided by total simulation area. Grey lines: ± standard deviation σ) were performed and analyzed with (red, empty squares, left axis) and without (green, empty circles, left axis) convolution with PSF. The molecular densities calculated from the resulting FCS curves of simulations are within 1σ with respect to the expected value. (TIF) [file pone.0121777.s006.tif]

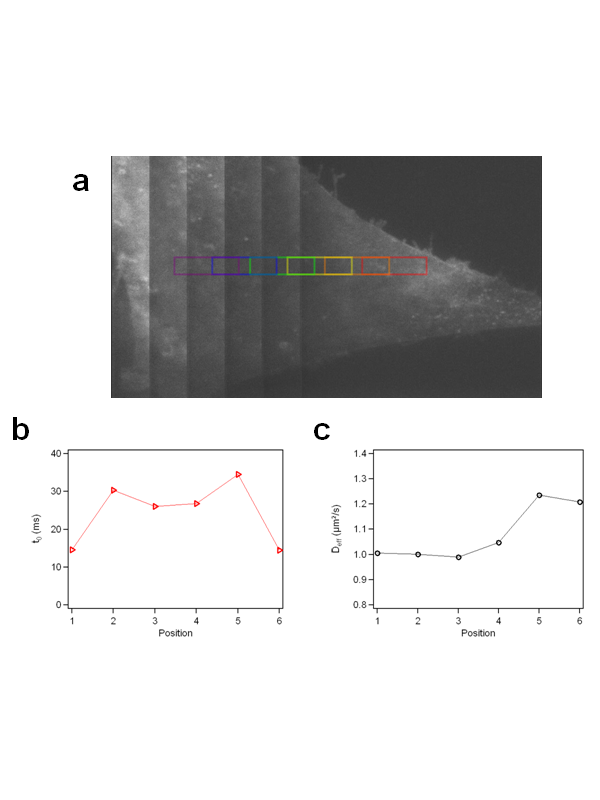

Supplement: S6 Fig — (a) TIRF camera images of the GPI anchored mGFP molecules in a PtK2 cell overlapped with 5 m shifts of the stage. Increasing wavelength rainbow colors indicate sequence of data acquisition for each ROI (100x20 pixels). (b) Time intercept values from the linear fit of the FCS law plots of bimFCS data, averaged over each ROI. (c) Effective diffusion coefficient from the linear fit of FCS law plots of bimFCS data. (TIF) [file pone.0121777.s007.tif]

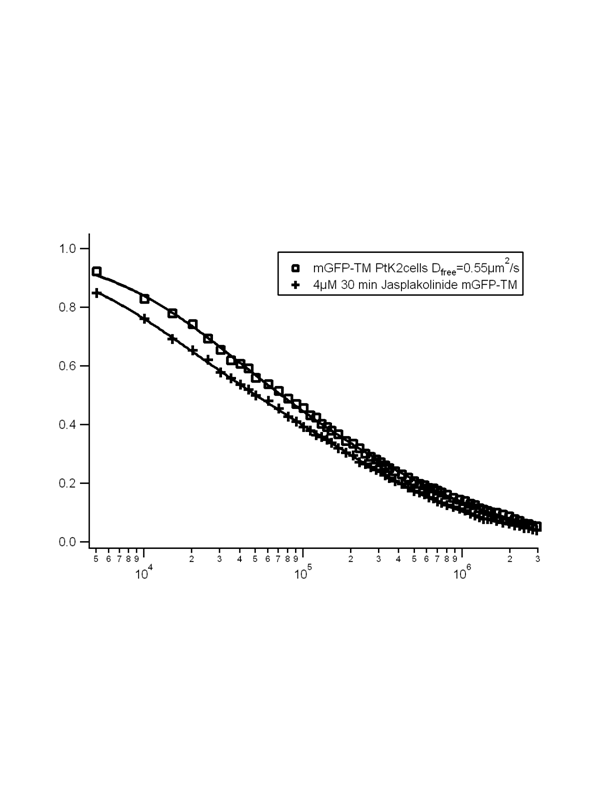

Supplement: S7 Fig — Normalized FCS curves (ω 2 = 0.087 μm 2) obtained from mGFP-TM (empty squares), and of mGFP-TM after incubating the cell with the actin filament disrupting drug Jasplakinolide treatment (plus signs), in PtK2 cells at 37°C. Data is fitted with two diffusion component square-pinhole FCS function. The original FCS curve clearly shows two diffusion coefficients (χ 2 doublefit/χ 2 singlefit = 0.039). After Jasplakinolide, the shape of FCS curve approximates that of free Brownian motion (χ 2 doublefit/χ 2 singlefit = 0.109), confirming that disruption of actin filaments removes the slower long-distance diffusion of the transmembrane protein. (TIF) [file pone.0121777.s008.tif]

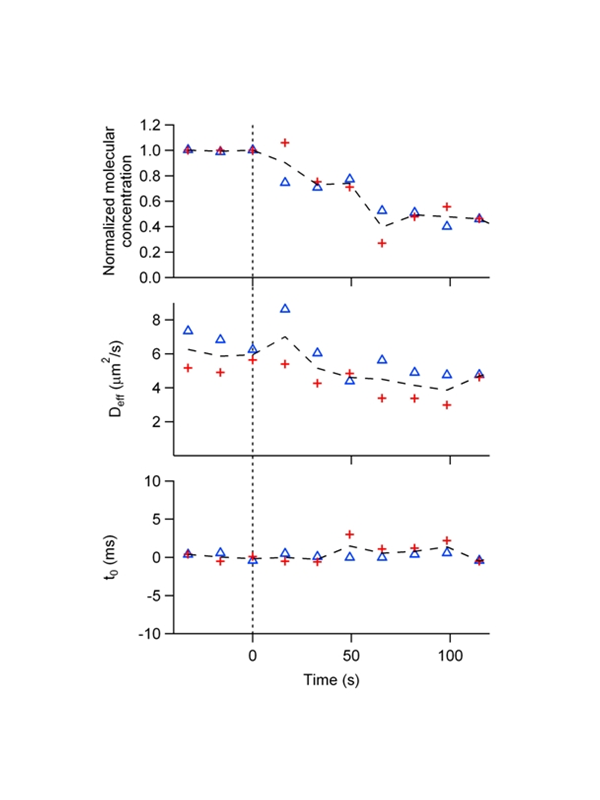

Supplement: S8 Fig — Changes of molecular concentration (top), effective diffusion coefficient (middle), and time axis intercept (bottom) of Liss-Rhod-PE diffusing in a supported lipid bilayer in response to the addition of αRhodamine antibody (2μg/ml) are shown. Time courses obtained from two separate experiments are shown (red plus sign and blue empty triangle), with the average between the two plotted in black dashed line. The normalized diffuser concentration dropped by around 50%. The t 0 value stayed around 0 throughout the time course. The effective diffusion coefficient of Liss-Rhod-PE dropped to about 70% of its starting value upon αRhodamine antibody treatment, compared to 50% in the case of mGFP-GPI dimerization. (TIF) [file pone.0121777.s009.tif]

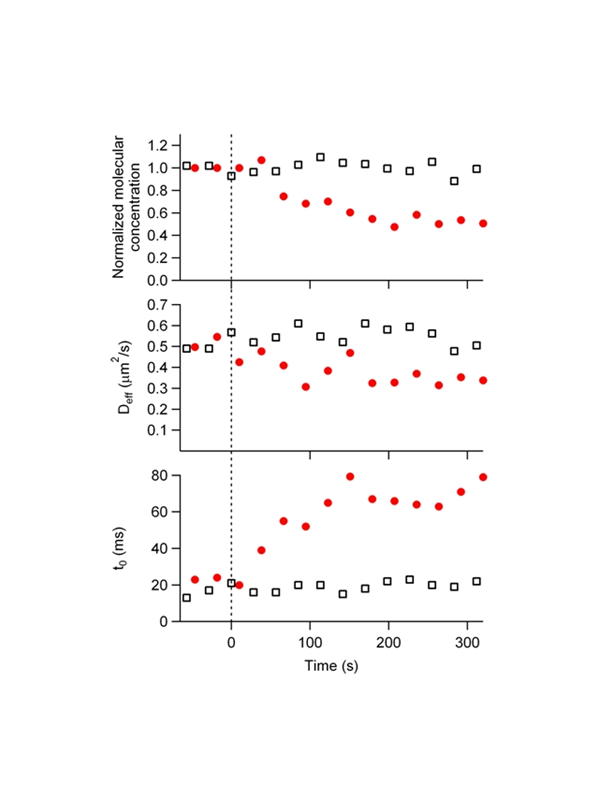

Supplement: S9 Fig — Changes of molecular concentration (top), effective diffusion coefficient (middle), and time axis intercept (bottom) of eGFP-GPI in response to the addition of αGFP antibody are shown (red, filled circles) together with the respective control traces (black, empty squares). Monoclonal αGFP antibody was added at t = 0 to a final concentration of 2μg/ml, while the same amount of buffer without antibodies was added to the control cell. Similar to the results from mGFP-GPI, the molecular concentration of eGFP-GPI dropped by 50%. This confirms effective dimerization induced by antibody cross linking. The effective diffusion coefficient of eGFP-GPI dropped to about 60% of its starting value, and the t 0value increased by three fold. (TIF) [file pone.0121777.s010.tif]

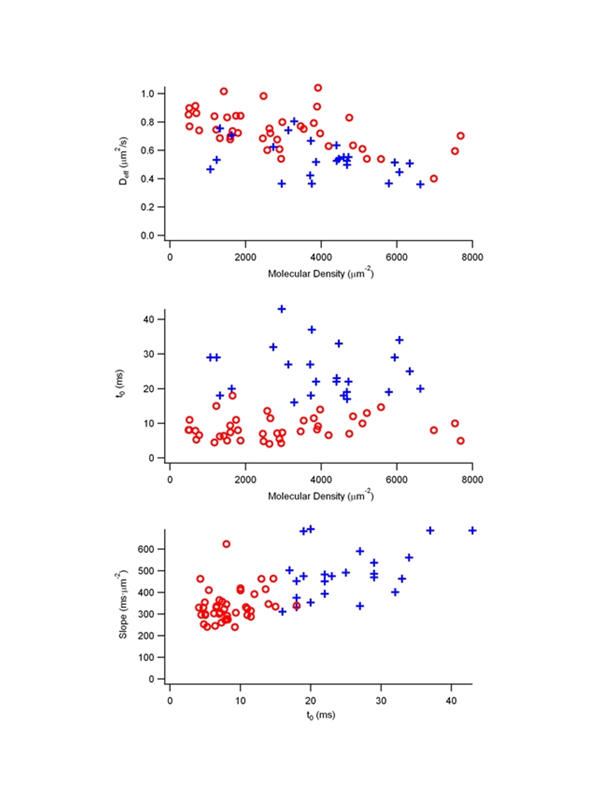

Supplement: S10 Fig — Effective diffusion coefficient (top) and time axis intercept (middle) obtained from mGFP-GPI (red, empty circles) and eGFP-GPI (blue, plus signs) are plotted as a function of measured molecular concentration. A slight negative correlation is found between Deff and molecular concentration for both mGFP-GPI (Pearson coefficient ρ p = -0.52) and eGFP-GPI (ρ p = -0.44), while t 0 is not dependent on molecular density. The correlation between t 0 and Deff is represented by plotting the slope of the linear fit, which is mathematically equal to 1/4Deff, versus t 0 for each measurement (C). The Pearson coefficient between slope and t 0 is 0.13 for mGFP-GPI and 0.44 for eGFP-GPI. bimFCS can be successfully performed on GFP labeled proteins expressing between 400 and 8,000 molecules/m2, more than an order of magnitude. Below this, the fluctuations are too small and rare compared to background and above this concentration, the fluctuations are too small of a fraction of the total signal. Using a brighter fluorophore, one can reach low concentrations, down to 50 molecules/m2 for Rhodamine. Over this range, we find little correlation between concentration and of D eff or t 0 or indicating that the heterologously protein expression does not affect the cell membrane ultrastructure. (TIF) [file pone.0121777.s011.tif]
