# Supplementary material for: Effect of Receptor Dimerization on Membrane Lipid Raft Structure Continuously Quantified on Single Cells by Camera Based Fluorescence Correlation Spectroscopy
Source: PLoS One. 2015 Mar 26;10(3):e0121777. doi: 10.1371/journal.pone.0121777 (PMC4374828; doi:10.1371/journal.pone.0121777)
Supplement: S1 Table — Parameters used in the Monte-Carlo Simulation of bimFCS of tracers diffusing on a regular grid with membrane fences and local traps. (DOCX) [file pone.0121777.s012.docx]

**Table S1 Description of Simulation Parameters**

| **Parameter description** | | | **Symbol** | | | | **Value / Range of values / Calculation** | |
| --- | --- | --- | --- | --- | --- | --- | --- | --- |
| *Monte Carlo parameters* | | | | |  | |  | |
|  | Lattice step size | | |  | | $1nm$ | |  |
|  | Simulation box | | |  | | $2\mu m\times2\mu m$ | |  |
|  | Observation area | | |  | | $1.6\mu m\times1.6\mu m$ | |  |
|  | Total simulated time | | |  | | $>40 sec$ | |  |
|  | Number of molecules in frame for FCS | | |  | | $100\pm20$ | |  |
| *Parameters to match simulation to experimental conditions* | | | | | | | | |
|  | Free diffusion coefficient | | | | $D^{out}$ | | in supported lipid bilayer $2.5{{\mu m}^{2}}/{sec}$;  on cells $1.3{{\mu m}^{2}}/{sec}$ | |
|  | 2D Gaussian width of PSF | | | | $\sigma$ | | $108.8nm$ | |
|  | Camera pixel size | | | |  | | $64 nm$ | |
|  | Effective waist of detection spot | | | | $\omega$ | |  | |
|  | Camera frame time | | | |  | | 1.5ms; or 1000 MCS steps | |
|  | Number of molecules in each frame | | | |  | | $100\pm20$ | |
| *Specific parameters for nano-domains* | | | | |  | |  | |
|  | Edge length of domains | | | | $l$ | | $20-75 nm$ | |
|  | Fraction of area covered by domains | | | | $d$ | | $0.1-0.3$ | |
|  | Relative diffusion coefficient inside domains | | | | $D_{rel}$ | | $0.1-1$; ${D^{in}}/{D^{out}}$ | |
|  | Probability of entering a domain | | | | $P_{in}$ | | $1$ | |
|  | Probability of exiting a domain | | | | $P_{out}$ | | $0.01-0.04, 0.1-0.2$ | |
| *Specific parameters for membrane fences* | | | | |  | |  | |
|  | | Spacing of square-grid fences | | | $a$ | | $50-500 nm$ | |
|  | | Hopping over probability for molecules | | | $P$ | | $0.01-0.1$ | |
| *Simulation observables* | | | | |  | |  | |
|  | Partition of molecules into domains | | | | $\alpha$ | | (molecules inside domains) / total molecules | |
|  | Confinement strength | | | | $S_{conf}$ | |  | |
|  | Effective diffusion coefficient | | | | $D_{eff}$ | | $1/{(4*(slope of t_{D} versus \omega^{2}plot))}$ | |
|  | Time-axis intercept of the FCS diffusion law | | | | $t_{0}$ | | y-intercept | |
